# Knowledge and mindset on anaphylaxis management in children and teenagers among caregivers in Slovenia

**DOI:** 10.1186/2045-7022-5-S3-O4

**Published:** 2015-03-30

**Authors:** Eva Šoster Križnik, Ljubica Deisinger, Mirjana Maslar, Andreja Obermayer, Vesel Tina

**Affiliations:** 1 Children's Department, General Hospital Celje, Celje, Slovenia; 2 Outpatient Pediatric Clinic for Allergology and Pulmonology, Ankaran, Slovenia; 3 Department of Allergology, Rheumatology and Clinical Immunology, University Children's Hospital, University Medical Center, Ljubljana, Slovenia

## Background

Appropriate first-line management of anaphylaxis is based on its recognition and application of intramuscular adrenaline as soon as possible. Parents, other caregivers including kindergarten and school personnel and also children themselves are first-line actors in case of anaphylaxis in a child or teenager and therefore comprehensive and thorough training for them is essential.

## Methods

We have applied ten short questions to parents and other caregivers (including kindergarten and school personnel) considering ability of their anaphylaxis recognition and management and willingness to help in case of anaphylaxis. The same questionnaire was used before and after 2-hour long course on recognition and management of anaphylaxis in three allergological centers in Slovenia.

## Results

138 participants were included. 75% of participants recognised correctly signs of anaphylaxis before and 74% after the course. Regarding the correct position during anaphylaxis there were 77% correct answers before the course and 99% after. The rate of correct answers about adrenaline being the most important drug during the anaphylaxis increased from 72% before the course to 100% afterwards. Regarding the correct order of management during the anaphylaxis there were 57% correct answers in the pre-course questionnaire and 94% in the post-course questionnaire. 33% of participants answered correctly the question about the correct application of adrenaline auto-injector Epipen before the course and 93 % after the course.

70% of participants would give adrenaline by auto-injector to the child with anaphylaxis before the course and 96% after the course. 29% of all participants were worried about the side effects of adrenaline, 34% of all about the legal consequences and 20% about the injury of a child with a needle. 43% of the participants believed public awareness campaign would contribute to better management of anaphylaxis in children, 34% of all thought the regulation of legislation is needed. Only 13% felt competent to help the child with anaphylaxis before the course but after the course that percentage raised to 71%.

## Conclusions

The 2-hour courses on anaphylaxis management enhanced theoretical ability and the willingness of appropriate first-line management of anaphylaxis in caregivers. Recognition of symptoms and signs of anaphylaxis was more difficult to teach. If the adrenaline auto-injector is prescribed to a child or teenager such trainings should be provided.

